# Quantitative Assessment of the Log-Log-Step Method for Pattern Detection in Noise-Prone Environments

**DOI:** 10.1371/journal.pone.0028107

**Published:** 2011-12-12

**Authors:** Florian Gomez, Ruedi Stoop

**Affiliations:** Institute of Neuroinformatics, ETH Zurich and University of Zurich, Zurich, Switzerland; Technical University of Madrid, Italy

## Abstract

Staircase-like structures in the log-log correlation plot of a time series indicate patterns against a noisy background, even under condition of strong jitter. We analyze the method for different jitter-noise-combinations, using quantitative criteria to measure the achievement by the method. A phase diagram shows the remarkable potential of this method even under very unfavorable conditions of noise and jitter. Moreover, we provide a novel and compact analytical derivation of the upper and lower bounds on the number of steps observable in the ideal noiseless case, as a function of pattern length and embedding dimension. The quantitative measure developed combined with the ideal bounds can serve as guiding lines for determining potential periodicity in noisy data.

## Introduction

The detection of patterns against a noisy signal background is a particularly important task for engineering and neuroscience [1]–[7]. Traditional approaches like Fourier analysis quickly break down under these conditions, or are far too ambiguous to be helpful from first principles (template-matching methods). Here, we assess the usefulness of an auxiliary tool. By providing information on the length and on metric aspects of putative patterns enclosed in a time series, the tool can guide the search for patterns. Although earlier [8], [9] the power of this method has been exemplified, so far no quantitative overview on its efficacy could be provided. In the present contribution, we introduce such a quantification.

Given a time series 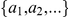 embedded in m-dimensional space using the standard *coordinate-delay construction* 
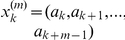
 [10]–[14], in the log-log plot of the correlation integral 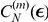


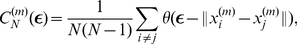
(1)instead of a straight line needed for the evaluation of the fractal dimension and correlation [15]–[18], steps may emerge. These steps emerge if the embedded points follow a simple generating pattern. Simple generating patterns lead to clusters of points in the embedding space that, in turn, lead to a sudden increase in the log-log plot of the point densities. This can be seen by choosing a random reference data point. Around this point, we enlarge the neighborhood radius 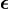, counting the points that fall into this neighborhood. After reaching a cluster of points, the count 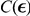 quickly increases with 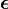, which leads to a step-like structure in the plot of 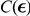.

Given a time series generated from a noise-free pattern of length 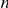 and using the maximum norm, these steps are sharp, and the number of steps visibly decreases with 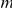. From the way how these steps propagate through the different embedding dimensions, we are able to derive upper and lower bounds to the observable number of steps appearing under ideal conditions as follows:

For 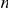 odd, the lower bound 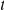 and the maximal number 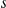 of steps have the expression
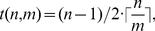
(2)

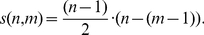
(3)


For 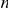 even, the lower bound 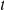 and the maximal number of steps 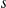 have the form
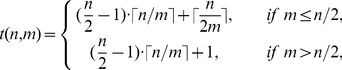
(4)and
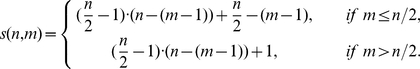
(5)


These results extend and detail insights from previous approaches [9].

**Figure 1:**
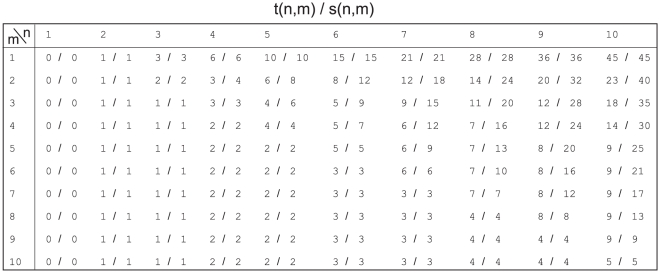
Ideal case. Lower bound 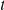/maximally observable steps 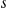, as a function of pattern length 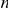 and embedding dimension 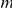.

By searching for steps, we can not only pin down data that are likely to contain patterns. With the help of the table presented in Fig. 1, we can also infer the length of putative patterns.

## Results

### Method validation

To what extent is the method reliable? In realistic time series, especially in neuroscience, a regular signal will be contaminated by jitter and noise. Jitter is commonly defined as the addition of an amount of signed (or unsigned) noise to the signal. Under its influence, a period-three signal of interspike intervals (ISIs) 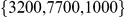 may change into a time series such as 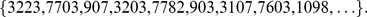 For this example, we added a jitter of 10 percent of the smallest ISI to the data, drawn from a uniform probability distribution. Alternatively, Gaussian or long-tailed distributions can be considered, which leads, in the range of interest, only to negligible differences. Noise is implemented by choosing a given percentage of the ISIs according to some random probability distribution. This can be achieved in two manners that reflect different ways of how the regularity-generating network is linked to the noise-generating part of the network: a) We can choose the next signal event with a probability 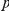 from the regular pattern and with a probability 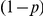 from the random distribution. b) Alternatively, with probability 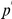 the whole regular pattern of length 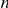 provides the 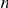 next signals, whereas with probability 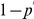 the signal event is drawn from the random distribution (for a fair comparison among the different paradigms, the probabilities must be rescaled as 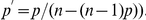 Motivated by neuroscience applications, here we focus for our results on the second paradigm.

**Figure 2:**
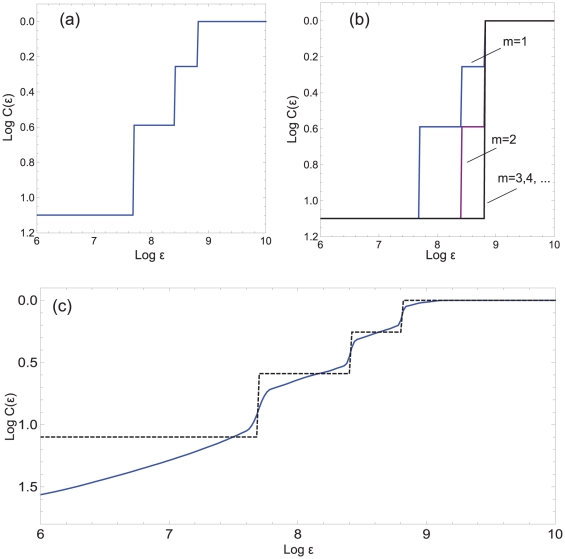
Log-log plots from a pattern of length 3. a) 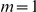, b) for increasing embedding dimension 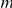, c) 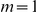, modification introduced by the presence of 20 percent jitter and 30 percent noise (pattern 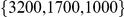.)

Upon the addition of jitter and noise, the steps gradually smear out and finally may no longer be visible. An example of a log-log plot displaying a step-like behavior is shown in Fig. 2. The following analysis focuses on a pattern of length 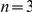. The analysis has, however, also been performed for patterns of length 5 and partially for length 7, with comparable results. Longer patterns have obtained little attention in experimental time series [19], [20].

In the log-log plot, jitter predominantly smoothens out the steps, whereas noise decreases the heights as well as the slopes of the stairs. We assess the ability of our method to highlight regular patterns of length 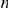 in jitter and noise contaminated data with the help of three criteria: a) How well can the predicted decrease of the number of steps with the embedding dimension 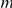 be evidenced? b) How well can exactly 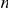 steps in the embedding dimension 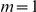 be detected? c) How well is a plateau, the flat part of the graph prior to the step, at embedding dimension 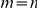 expressed if compared to that observed at 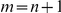 [9]?

For the first criterion, we verified whether the predicted decrease of the number of steps as a function of 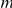 was observed or not. To this end, we tested whether a single vertical step was visible at 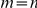. For this we preset three height levels 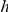 with corresponding quality weights 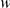 (denoted 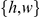) 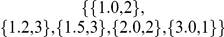 that in the ideal case the derivative of the log-log plot would all exceed. Given a particular preset height level, we rewarded the detection of exactly one peak in the derivative (corresponding to a sharp step-like increase in the original plot) with the level's corresponding weight and used the resulting sum over the height levels as ‘quality’ measure. Added noise, however, may trigger a reappearance of the theoretically vanishing steps at and beyond the embedding dimension at which only one step should emerge. To eliminate this problem, if two or three steps emerged in the data, we compared the time series vs. surrogate (i.e. randomly permuted) series, in which the repeated steps emerge most pronouncedly. To characterize the distance from the surrogate case, the quality of the time series data was subtracted from the surrogate quality. The final ‘quality’ measure was thus composed as sum of a first measure for the visibility of exactly one step and a second term which, being nonzero only in the case of two or three observed steps, reflects the distance from the surrogate case.

For the second criterion, in order to quantify the visibility of exactly three derivative peaks at 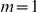 we proceeded with a peak-detection algorithm as in criterion (a) yet with a slightly different attached level-weight-vector 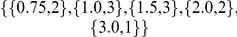. For the third criterion, the plateau flatness at 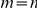 was compared to 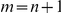. A plateau was counted, if the derivative of the plot was below values 
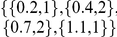
 (again with corresponding weights 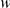, 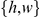). The weighted average counts obtained for 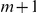 were then subtracted from the weighted average counts obtained for 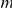.

For all criteria assessments (a), (b) and (c), we approximated the derivative values as difference quotients between two consecutive data points, for which 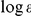 was increased in steps of 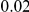. Certainly the above described algorithms are not the unique possibility to reasonably quantify the proposed criteria. We however argue that the algorithms, together with the carefully selected weight vectors, do provide a measure which is in accordance with the human eye's perception of peaks and plateaus.

**Figure 3:**
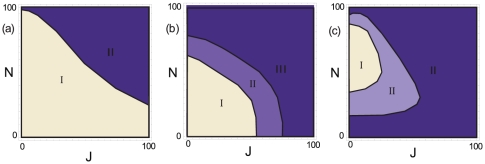
Approximate phase boundaries, for noise 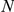 and jitter 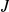 in units of percents of events in the data and in percents of the smallest interval in the pattern. Fulfillment of the criteria is expressed by three degrees: Region I: excellent, region II: fair, region III: ambiguous. a) Measure for the decrease in steps with 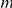 (only two regions: I and III). b) 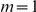-criterion; c) difference in plateau visibility for 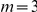 compared to 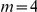. Regions I, II and III as in a).

By dividing through the observed maximal measure, the three measures were normalized and a contour-plot with suitable contours was drawn. Fig. 3 shows the results obtained. We defined two or three regions of various visibility for each of the criteria. Not surprisingly, the visibility of exactly 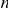 peaks for 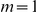 (Criterion (b)) is best in the case of little noise and little jitter. Nevertheless, the visibility is considerably good for noise fractions up to 50 or 60 percent. It is natural, however, that results would be worse in the case of longer patterns or steps being more closely located. Clearly, the seven steps of a length-7 pattern are more difficult to distinguish since with increasing jitter the peaks in the derivative may overlap. Criteria (a) and (c) are what we consider to be the strongest indicators for the occurrence of patterns. The emergence of the “natural” situation 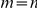 - where patterns are completely inserted but no additional terms spoil the characteristic behavior - is most helpful in the case of little jitter but high noise values. In regions of up to 90 percent of noise, when all other methods normally fail, the plateau occurring at 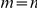 compared to 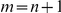 reliably indicates a pattern of length 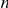. We tested criterion (c) for a generic pattern of length 5 comparing the dimensions 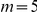 and 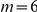 using exactly the same algorithm. Even though there are theoretically two visible steps in this case, the two plateaus quickly merge into a single one. The resulting plot looks very similar with even a slightly extended range of visibility. We thus suppose criterion (c) to be fairly independent of the underlying pattern length. In regions where the criterion (c) fails, i.e. for little noise and high jitter, criterion (a) may serve as indicator of the pattern length. The visibility of one single step in dimension 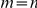 alone yet does not prove a pattern length 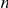, since patterns of length 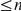 may also lead to such a single step. Comparing to the embeddings 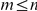 where more steps should occur helps to exclude these cases. Moreover, high jitter values may merge two steps, if these steps are close together. The possible overlap of neighboring steps thus sets the natural limit to the method. Yet this happens only in the case of highly jittered signals or specific patterns having two distinct distances very close together. In the latter case, nonetheless still a pattern will be indicated, albeit of the wrong length.

### Proof of the analytical formula for 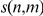 and 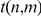


**Figure 4:**
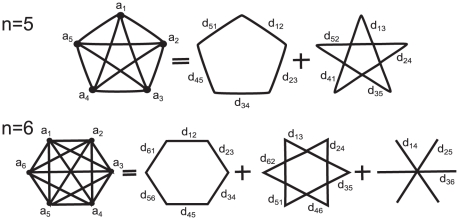
Graphs of componentwise distances. Decomposition of potential distances in the maximum norm for odd and for even pattern lengths 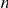 into nearest-, next-nearest-, etc., neighbor subgraphs. Each subgraph can be treated separately.

For a proof of (2)–(5), we decompose the graph of componentwise distances 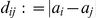 into subgraphs connecting nearest-, next-nearest-, etc. neighbors, see Fig. 4. The idea underlying the optimized proof with sharper bounds is, as in the old proof in [9], the following: The choice the maximum norm makes is restricted to consecutive 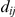
's on one distinct subgraph. For 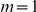, every ‘comparison’ yields a winner, hence we have 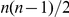 steps. For larger 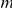, the ordering of 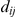 on the subgraphs is crucial. When 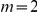 and 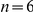, a monotonous ordering 
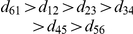
 yields 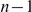 steps; in 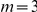, 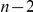 steps, and so on. Contrarily, if we have a ‘regular’ distribution of the biggest three distances 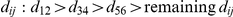, only 3 steps are contributed when 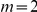. For odd 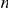, each subgraph follows the rules for the monotonous ordering of a maximal number, from where we get 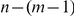 steps, and for a regular ordered set 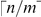 steps. From this, we arrive at 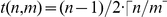 and 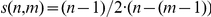. For even 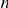, 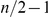 subgraphs follow the same rules as above, except for the one with 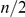 lines, which only contributes one step if 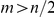.

## Discussion

To summarize, we emphasize the remarkable performance of the method under very noisy conditions. As a general advice (generally true for time series analysis!) we propose not to rely on one single criterion, but to combine all aspects to obtain a coherent picture. The reader may thus derive an overall goodness-of-method measure by adding the measures obtained from the different criteria. This might help to *a priori* evaluate the applicability of the method to a user's problem. As guideline for the practical use of the method, we suggest to embed a given time series in spaces of multiple dimensions 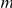 up to 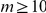 in order to capture possible pattern lengths of such order. Regarding the computation of the correlation integral, it is important to sample densely enough among randomly selected reference points (e.g., for 10.000 data points, we recommend something above 200 reference points). Equipped with the log-log curves for multiple 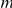, a significant plateau flatness difference between to consecutive 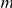 (criterion (c)) can serve as first indicator of the pattern length [9]. Criteria (a) and (b) may be helpful to confirm such a suspicion and to gain additional, metric information about the pattern. Moreover, the slope of the lines in the step-free regions may give interesting insights into the fractal dimension of a possible attractor.

## Materials and Methods

All computations were performed in a C++ and *Mathematica* environment on a custom laptop. The method validation was based on the length-3 pattern 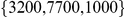. The correlation integral was evaluated for a total of 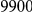 embedded points, where 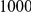 points were used as reference points. For a total of 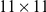 jitter-noise-configurations (from 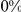 to 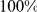 in steps of 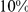), we evaluated the three described criteria. A set of levels appropriate for the classification into the ‘excellent’, ‘fair’ and ‘ambiguous’ evaluation regimes resulted in the contour-plots shown in Fig. 3.
